# Pathophysiology of Portal Hypertension and Esophageal Varices

**DOI:** 10.1155/2012/895787

**Published:** 2012-05-15

**Authors:** Hitoshi Maruyama, Osamu Yokosuka

**Affiliations:** Department of Medicine and Clinical Oncology, Chiba University, Graduate School of Medicine, 1-8-1, Inohana, Chuo-ku, Chiba 260-8670, Japan

## Abstract

Esophageal varices are the major complication of portal hypertension. It is detected in about 50% of cirrhosis patients, and approximately 5–15% of cirrhosis patients show newly formed varices or worsening of varices each year. The major therapeutic strategy of esophageal varices consists of primary prevention, treatment for bleeding varices, and secondary prevention, which are provided by pharmacological, endoscopic, interventional and surgical treatments. Optimal management of esophageal varices requires a clear understanding of the pathophysiology and natural history. In this paper, we outline the current knowledge and future prospect in the pathophysiology of esophageal varices and portal hypertension.

## 1. Introduction

Esophageal varices are the major complication of portal hypertension. It is detected in about 50% of cirrhosis patients, and approximately 5–15% of cirrhosis patients show newly formed varices or worsening of varices each year [1–5]. It is a hemodynamic abnormality characterized by sudden bleeding episode; about a third of all patients with esophageal varices show bleeding episode [6].

 A key objective in managing the cirrhotic patients having varices is the primary prevention of bleeding. Either nonselective *β*-blockers or endoscopic variceal ligation is the treatments of choice for the primary prevention of variceal bleeding [7]. Patients who survive an episode of variceal bleeding are at high risk for rebleeding. Combination of *β*-blockers and band ligation is the preferred therapy to reduce rebleeding rate [7]. Failures of medical treatment should be managed aggressively with transjugular intrahepatic portosystemic shunting (TIPS), preferably using expanded polytetrafluoroethylene (ePTFE) covered stents [7]. Because of higher rates of morbidity and mortality, rescue derivative surgery should only be considered in low-risk patients.

Optimal management of esophageal varices requires a clear understanding of the pathophysiology and natural history. In this paper, we outline the current knowledge and future prospect in the pathophysiology of esophageal varices and portal hypertension.

## 2. Pathophysiology of Portal Hypertension and Esophageal Varices

Portal hypertension is associated with both increased portal inflow and increased outflow resistance [8]. Although direct measurement of portal pressure may provide accurate condition, an invasiveness of portal venous catheterization limits the clinical application. Hepatic venous catheterization is the most common technique to determine the portal pressure. Wedged hepatic venous pressure (WHVP) reflects sinusoidal pressures, and hepatic venous pressure gradient (HVPG) is the difference between WHVP and free hepatic venous pressure, being a good predictor for the severity of portal hypertension. Portal hypertension results in the development of collateral vessels, which are the route blood returning to the systemic circulation from portal system bypassing the liver.

## 3. Natural History and Bleeding Risks of Esophageal Varices

Varices may not develop and bleed when the HVPG is lower than 12 mmHg [5, 9]. That is, varices are closely associated with the condition of HVPG higher than 12 mmHg. Red sign and variceal size (medium to large grade) on endoscopy are representative for bleeding risk of esophageal varices [7, 10]. Severity of liver function reserve and presence of ascites are also important risk factors for variceal bleeding [10].

 The bleeding risk decreases over time from the time that varices are identified; most bleeding episodes occur within the first 2 years after identification of varices [6]. Once bleeding occurs, spontaneous cessation of bleeding occurs in only up to 40% of individuals, and the bleeding is associated with the mortality of 20% or more at 6 weeks [11, 12].

 Patients who survived an episode of acute variceal hemorrhage have a high risk of rebleeding and death [13]. The median rebleeding rate in untreated individuals is around 60% within 1-2 years of the index bleeding, with a mortality of 33% [14, 15]. Therefore, care should be taken to prevent recurrent bleeding prior to discharge from the hospital for patients who have recovered from an episode of variceal bleeding.

 Patients with an HVPG > 20 mmHg measured within 24 hours of variceal bleeding have been identified as being at a higher risk for early rebleeding or failure to control bleeding (83% versus 29%) and a higher 1-year mortality (64% versus 20%) compared to those with lower pressure [16, 17]. Large varices, age over 60 years' old, renal failure, and severe initial bleeding as defined by a hemoglobin <8 g/dL at admission, are the risk factors for early rebleeding [6].

## 4. Pathophysiology of Portal Hypertension and Esophageal Varices

### 4.1. Hepatic Vasodilators

**(1) Nitric Oxide**

Nitric oxide (NO) is a powerful endogenous vasodilator (Table 1), and it modulates the intrahepatic vascular tone [18]. NO is produced from the amino acid l-arginine by NO synthases. It is the natural ligand for soluble guanylate cyclase and is responsible for an increase in the levels of cyclic guanosine monophosphate, the final agent responsible for the relaxation of the vascular wall through the extrusion of cytosolic Ca^2+^.

NO inhibition increases portal pressure in isolated perfused rat livers, and the hepatic response to norepinephrine is markedly enhanced after NO inhibition, suggesting a role of NO in modulating hepatic vascular tone in normal conditions [18]. However, in the cirrhotic liver, the synthesis of NO is insufficient to compensate for the activation of vasoconstrictor systems frequently associated with cirrhosis. This occurs despite a normal expression of eNOS (endotherail NO synthase) mRNA and normal levels of eNOS protein [19], and the decreased activity of hepatic eNOS in cirrhosis is due in part to increased expression of caveolin [20]; as Akt-induced phosphorylation of eNOS reverses inhibitory conformation of eNOS in association with caveolin-1 [21].

The insufficient hepatic NO production may account for the increased intrahepatic vascular resistance in cirrhosis, thereby worsening portal hypertension. These findings may be supported by the data; the infusion of l-arginine, the precursor of NO biosynthesis, and the administration of nitrates (exogenous donors of NO) decrease portal pressure. Further, enhancement of the expression of NO synthase in liver cells, through the portal injection of adenovirus coupled with the gene encoding NO synthase, significantly reduces portal pressure.

Recent study has shown that myr-Akt gene therapy restored Akt activation and NO production in the cirrhotic liver, suggesting the potential availability of alternative treatment for portal hypertension [22]. The other study reported that simvastatin stimulated hepatosplanchnic output of NO products and decreases hepatic resistance in cirrhosis due to the increased Akt-dependent endothelial NO synthase phosphorylation [23]. The data was supported clinically by the randomized controlled trial [24]. NO also promotes apoptosis of hepatic stellate cell through a signaling mechanism that involves mitochondria, is mediated by reactive oxygen species, and occurs independent of caspase activation [25]. This NO-dependent apoptosis, which may maintain sinusoidal homeostasis, is expected as a future treatment of portal hypertension.

**(2) Carbon Monoxide**

Carbon monoxide (CO), a by-product of heme group oxidation by heme oxygenases (HOs), is considered as an important modulator of intrahepatic vascular resistance [26]. CO activates guanylate cyclase and thereby promotes smooth muscle relaxation, in spite of being less potent than NO. The inhibition of CO production increases portal resistance in normal livers, and HO/CO system is activated in patients with liver cirrhosis. In addition, plasma CO levels directly correlated with cardiac output and inversely with systemic vascular resistance and mean arterial pressure. Thus, CO may be closely related to the hyperdynamic circulatory state in cirrhosis [27].

### 4.2. Splanchnic Vasodilatation

Portal venous inflow tends to increase in cirrhosis, particularly in advanced stages of portal hypertension, due to the vasodilatation in the splanchnic organ. This increased blood flow is one of the key factors which contribute to the pathophysiology of portal hypertension [28]. There are some possible mechanisms which account for the portal hemodynamic abnormalities, neurogenic, humoral, and local mechanisms; vasodilators in the systemic circulation have been examined to explain the pathophysiology of portal hypertension. Increased levels of vasodilators are observed because of impaired hepatic function or development of portosystemic collaterals, as most of them underwent hepatic metabolisms.

**(1) NO**

NO is involved in the regulation of splanchnic and systemic hemodynamics in portal hypertension. Excessive production of NO may be one of major reasons for the vasodilatation, as splanchnic vasoconstrictive effect caused by NO inhibitors in animal is significantly greater in portal hypertension model than control model [29]. An overproduction of NO has also been clearly demonstrated in vitro in perfused mesenteric artery preparations from portal hypertensive rats [18]. Furthermore, the fact that cirrhotic patients show increased levels of serum and urinary concentrations of nitrite and nitrate, which are products of NO oxidation, also supports a role of NO in the pathophysiology of portal hypertension [30]. An increased expression and an increased activity of eNOS account for the increased production of NO. Further, there are some factors which may activate the constitutive NO synthase: shear stress, circulating vasoactive factors (e.g., endothelin, angiotensin II, vasopressin, and norepinephrine), and overexpression of the angiogenic factor vascular endothelial cell growth factor (VEGF) [31]. Recent study suggests that mild increases of portal pressure upregulate eNOS at the intestinal microcirculation through VEGF upregulation [32].

**(2) Glucagon**

Glucagon is a humoral vasodilator which is associated with splanchnic hyperemia and portal hypertension. Two mechanisms are considered for vasodilation by glucagon; relaxing the vascular smooth muscle and decreasing its sensitivity to endogenous vasoconstrictors, such as norepinephrine, angiotensin II, and vasopressin [33]. Plasma glucagon levels are increased in cirrhotic patients and experimental models of portal hypertension, due to decreased hepatic clearance of glucagon as well as an increased secretion of glucagon by pancreatic *α* cells [34].

Administration of glucagon antibodies or somatostatin reverses the increase in splanchnic blood flow as a result of normalizing circulating glucagon levels. Additionally, concomitant infusion of glucagon blocks the response in portal hypertensive rat model, and increased circulating glucagon levels in normal rats to values similar to those observed in portal hypertension cause a significant increase in splanchnic blood flow [35, 36]. According to these data, hyperglucagonemia may be responsible for some part of the splanchnic vasodilatation of chronic portal hypertension. The role of glucagon in the splanchnic hyperemia of portal hypertension provides a rationale for the use of somatostatin and its synthetic analogs to reduce glucagon level, thereby treating portal hypertension [37].

**(3) Other Mediators**

CO is one of the vasodilators; an expression and activity of HO are increased in splanchnic tissues in portal hypertension [27]. HO also stimulates VEGF production, resulting in the development of hyperdynamic splanchnic circulation [38].

Recent study has shown that endocannabinoids have a significant role in the hyperdynamic circulation of portal hypertension [39]. Endogenous cannabinoid anandamide is increased in the monocyte fraction of blood from cirrhotic humans and rats, and also expression of the cannabinoid 1 (CB1) receptors is increased in hepatic human endothelial cells. It is considered that activation of endothelial CB1 receptors may stimulate NO production, though the mechanism is unclear. Therefore, inhibition of CB1 receptor blockade may have a possibility of treatment for portal hypertension as a result of reduction of portal flow.

Prostacyclin is an endogenous vasodilator produced by vascular endothelial cells [40]. It causes vascular smooth muscle relaxation by activating adenylate cyclase and augmenting the intracellular level of cyclic adenosine monophosphate. Two different isoforms of cyclooxygenase COX are involved in the biosynthesis of prostacyclin, COX1 and COX2. Both are involved in the increased prostacyclin production by the mesenteric vascular bed of portal vein-ligated rats and the selective inhibition of COX-2 and, to a lesser extent of COX-1, improve the endothelial-dependent vasodilatation in response to acetylcholine [41]. A partial reversal effect for splanchnic vasodilatation after COX blockade might be applicable to ameliorate the hyperdynamic circulation state and/or portal pressure in cirrhosis.

### 4.3. Hyperdynamic Circulation

The portal hypertension is directly related to portal inflow and/or outflow resistance, as determined by Ohm's law “portal pressure = portal venous inflow × outflow resistance.” Portal venous inflow is affected by hyperdynamic circulation, which is characterized by systemic and splanchnic vasodilatation, low systemic resistance, plasma volume expansion, and high cardiac index [8]. Splanchnic vasodilatation contributes to increasing substantial blood volume which returns to portal venous system. Peripheral vasodilatation activates endogenous neurohumoral systems that cause sodium retention, which leads to expansion of the plasma volume, followed by an increase in the cardiac index. Expansion of plasma volume is a necessary step to maintain an increased cardiac index, which in turn aggravates portal hypertension. This provides the rationale for using a low-sodium diet and diuretics in the treatment of portal hypertension.

### 4.4. Portosystemic Collateral Circulation

The development of portal-collateral circulation is one of the hemodynamic features of portal hypertension. Formation of collaterals is a complex process involving the opening, dilatation, and hypertrophy of preexisting vascular channels. Collaterals develop according to the increased portal pressure, and minimum threshold level of HVPG may be 10 mmHg for the development of portosystemic collaterals and esophageal varices [5, 9].

 The vascular resistance of collateral vessels may be a major component of the overall resistance to portal blood flow and, therefore, may be important in determining portal pressure. In addition, although it was traditionally thought that the hyperdynamic splanchnic circulation state associated with portal hypertension was the consequence of active splanchnic vasodilatation, recent data suggests that the increased neovascularization in splanchnic organs plays an important role in allowing the increase in splanchnic blood inflow [42]. In addition to the increased portal pressure, formation of portosystemic collateral vessels in portal hypertension is influenced by a VEGF-dependent angiogenic process and can be markedly attenuated by interfering with the VEGF/VEGF receptor-2 signaling pathway. This finding suggests that manipulation of the VEGF may be of therapeutic value.

 Although the factors which modulate the resistance of collateral vessels have not been clarified, NO may be one of the factors which regulate portal collateral vascular resistance [43]. Effects of isosorbide-5-mononitrate (IMN) and nitroglycerin (NTG) to reduce collateral resistance in cirrhosis may be associated with this NO function. These vessels are also probably hypersensitive to serotonin (5-HT), which markedly increases their vascular tone. In portal hypertensive animals, the administration of selective 5-HT2 receptor blockers decreases portal pressure.

### 4.5. Vasoconstrictors and Hepatic Vascular Bed

Endothelins (ETs) are a family of homologous 21 amino acid peptides which include ET-1, -2, -3, and -4. They exert various biological effects, vasoconstriction, and stimulation of cell proliferation in tissue. One of the major roles of ET is modulation of vascular tone in cirrhosis [44]. Two major receptors function to mediate, ET-A receptor and ET-B receptor. The former shows a high affinity for ET-1, not for ET-3, and mediates constriction, and the latter has equal affinity for ET-1 and ET-3. Activation of ET-B receptors located on the vascular smooth muscle cells promotes vasoconstriction, whereas activation of ET-B receptors located on endothelial cells promotes vasodilatation, which is mediated by enhanced NO and prostacyclin production by the endothelial cell.

 Plasma levels of ET-1 and ET-3 are increased in cirrhotic patients [45]. The level is dominant in patients with ascites. A net release of ET-1 and ET-3 in the splanchnic circulation has been observed in cirrhotic patients but not in controls, suggesting an increased production of ET-1 and ET-3. In fact, increased expression of ET-1 is reported in human cirrhotic livers [46]; endothelial cells, hepatic stellate cells (in their activated phenotype), and bile duct epithelial cells are the major intrahepatic sources of ET-1. However, the precise mechanism and role of ETs in increasing the vascular tone in cirrhosis remains unclear.

 Angiotensin II is a powerful vasoconstrictor, which may contribute to increasing hepatic resistance [47]. A-II antagonists, inhibitors of the converting enzyme, or A-II receptors blockers may have a potential to reduce portal pressure, though their effects may be accompanied with systemic hypotension.

 Norepinephrine is also a vasoconstrictor, which is involved in the regulation of hepatic vascular tone [48, 49]. The administration of *α*-adrenergic antagonists, such as prazosin, inhibits the increase of resistance by norepinephrine. In addition, the hepatic vascular bed of cirrhotic livers exhibits an exaggerated response to the *α*-adrenergic agonist methoxamine. This hyperresponse is associated with the overproduction of thromboxane A_2_ (TXA_2_) by COX-1 isoenzyme and is completely corrected by pretreating the livers with nonselective COX blockers, COX-1-selective blockers, or TXA_2_ antagonists. Therefore, an increased production of TXA_2_ markedly enhances the vasoconstrictive response of the cirrhotic hepatic vascular bed to methoxamine. It remains to be solved, however, that whether this effect is also shared by other vasoconstrictors.

### 4.6. Endothelial Dysfunction

The endothelium under normal condition has a function to produce vasodilators in response to increases in blood volume and blood pressure or to produce vasoconstrictors to prevent or attenuate the concomitant increase in pressure. However, abnormality in the endothelium-related vascular reaction occurs in several pathologic conditions, that is, endothelial dysfunction [50]. It is considered as one of the main mechanisms which account for the increased vascular tone observed in several vascular disorders, such as arterial hypertension, diabetes, and atherosclerosis, and have been attributed to a diminished NO bioavailability or to an increased production of endothelial-derived contracting factors, such as prostaglandin H_2_ (PGH_2_)/TXA_2_, ET, or anion superoxide [18]. The intrahepatic vascular bed in cirrhosis also exhibits endothelial dysfunction [51]. Indeed, studies performed both in cirrhotic patients and in experimental models have shown that, contrary to what happens in normal livers, the cirrhotic liver cannot accommodate the increased portal blood flow caused by the postprandial hyperemia, which determines an abrupt postprandial increase in portal pressure [52].

 Studies have shown that endothelial dysfunction is associated with an abnormal response to the endothelium-dependent vasodilator acetylcholine [51, 53]. This impaired response may be related to an increased production of TXA_2_ and completely prevented by selective COX-1 blockers and TXA_2_ antagonists. These data suggest that an increased production of a COX-1-derived vasoconstrictor prostanoids, probably TXA_2_, may be responsible for endothelial dysfunction [53].

 Recent studies have shown the possibilities of additional treatments; one is tetrahydrobiopterin, an eNOS cofactor, which increases eNOS activity and significantly improves the vasodilator response to acetylcholine in rats with cirrhosis [54]. It may have a potential role for the treatment of portal hypertension by improving the endothelial dysfunction. The other is “statins,” which decreases intrahepatic vascular resistance and improve flow-mediated vasodilation of liver vasculature in cirrhotic liver, due to increase of NO production and improvement of hepatic endothelial dysfunction [23, 24]. 

## 5. Conclusions

Many advances in the management of portal hypertension and variceal bleeding have occurred over the last 20 years. The key factor for variceal rupture is the wall tension of varices, which is determined by the “Lapace's law”: wall tension = (variceal pressure – luminal pressure) × radius/thickening of variceal wall. This tension is the force which is generated by the variceal wall opposing further distension. When the wall tension reaches the critical point of the elastic limit of the varices, rupture occurs. Red sign on endoscopy is a significant indicator to apply prophylactic treatment of esophageal varices. Effective primary prevention for variceal bleeding is now available by nonselective beta blockers or band ligation. Active bleeding should be managed with band ligation alone or combined with somatostatin or octreotide; TIPS and surgery may be positioned as salvage therapy for those who fail endoscopic treatment. Survivors of a variceal bleed should be evaluated for liver transplant.

**Figure 1:**
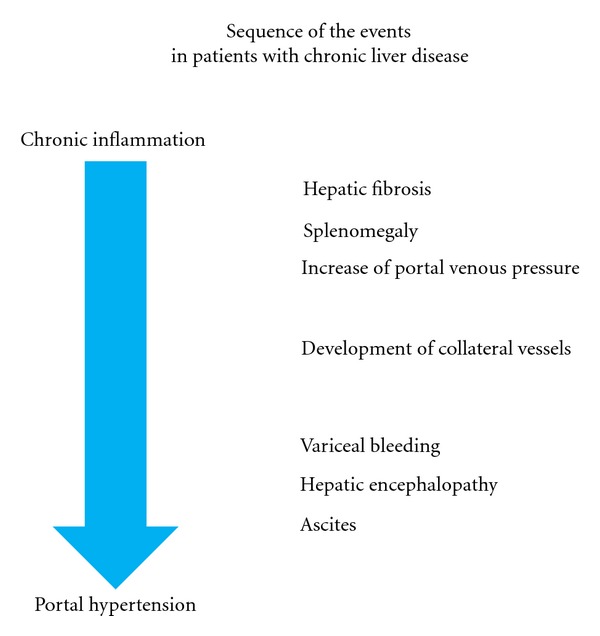
Sequence of the events in patients with chronic liver diseases. Possible events are listed from chronic inflammation to portal hypertension.

 Since the occurrence of clinical events due to portal hypertension is related to the hemodynamic changes (Figure 1), the goal of long-term pharmacologic therapy in patients with portal hypertension should be a reduction of the HVPG by at least 20% from baseline values and preferably to below the threshold of 12 mmHg. This may explain some of the interindividual variability in hemodynamic response to pharmacological treatment. Recent study has shown that rifaximin may have a possibility to decrease risk of variceal bleeding, and the other complications related to portal hypertension [55]. The pathophysiology in portal hypertension is likely to be multifactorial in origin; various interactive regulations may be present to compensate for the effect of vasoactive mediators. It is a continuous challenge to unveil the mechanism and to develop more effective therapeutic measures.

## Figures and Tables

**Table 1:** Vasoactive mediators.

Vasodilators	Vasoconstrictors
Nitric oxide	Endothelin
Carbon monoxide	Angiotensin II
Glucagon	Norepinephrine
Endocannabinoid	Vasopressin
Prostaglandin	
